# Structural and nonstructural protein divergence correlates with strain-specific vector competence for Zika virus

**DOI:** 10.1186/s13071-026-07569-w

**Published:** 2026-07-13

**Authors:** Rhoda Ntim Owusu, Vijay P. Chelikani, Millicent Sifuna, Melanie Bretton, Justice Libertyann Ward-Polonski, Kwasi Baako Antwi, Lucia Kiio, Aurelien Vigneron, Maria Gorreti Onyango

**Affiliations:** 1 Department of Biological Sciences, Texas Tech University, 2901 Main St, Lubbock, TX 79409 United States; 2 Department of Mechanical Engineering, Texas Tech University, 41021, Lubbock, TX 79409 United States; 3 Laboratoire d’Ecologie Microbienne, UMR CNRS 5557, UMR INRAE 1418, Universite Claude Bernard Lyon 1, VetAgro Sup, Villeurbanne, France

**Keywords:** Zika virus, *Aedes aegypti*, Viral lineage, Transcriptomic profiling

## Abstract

**Background:**

Zika virus (ZIKV) emergence in the Americas was driven by Asian-lineage strains, whereas African lineages represent the ancestral form of the virus and remain understudied in terms of transmission potential. Whether genetic differences between viral strains influence their ability to infect and be transmitted by American *Aedes aegypti* mosquitoes remains unclear. We investigated strain-specific differences in vector competence, host transcriptional responses, and viral genomic variation.

**Methods:**

Zika virus strains representing Neotropical (Honduras) and Old-World (Senegal) lineages were orally administered to *Ae. aegypti* mosquitoes via infectious blood meals. Vector competence was assessed at 14 days post-infection by quantifying infection, dissemination, and transmission using RT-qPCR targeting the NS1 gene. RNAseq was performed on infected mosquitoes to evaluate strain-associated transcriptional responses, followed by differential expression analysis.

**Results:**

Dissemination was markedly higher for the Neotropical strain, as was transmission. Viral loads were significantly higher in whole bodies, legs, and saliva of Neotropical-infected mosquitoes. Neotropical infection suppressed immune- and barrier-associated genes. We observed nucleotide divergence between isolates; the Neotropical strain harbored three nonsynonymous substitutions (Envelope, NS1, NS2A), whereas the Old-World strain retained a single NS2A substitution. Infection did not alter secondary blood-feeding success but significantly reduced fecundity especially among the population exposed to the Neotropical strain.

**Conclusions:**

The evaluated Neotropical and Old-World ZIKV isolates exhibit markedly different transmission efficiencies in Mexican *Ae. aegypti*. Limited amino acid variation in structural and replication-associated proteins, together with strain-specific host gene modulation, may contribute to the enhanced transmission potential of the Neotropical isolate. These findings highlight specific viral genetic backgrounds as critical determinants of intra-vector dynamics and transmission risk.

**Graphical Abstract:**

**Figure:**
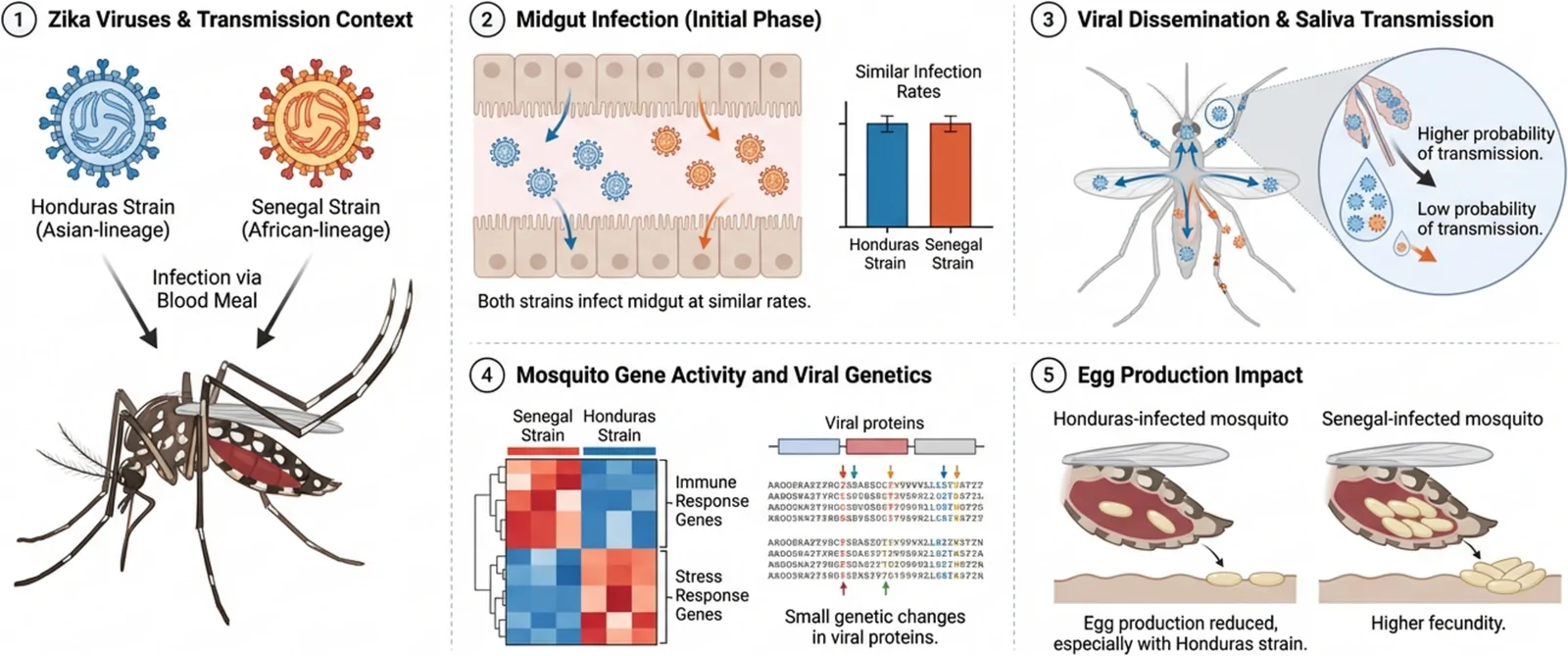

**Supplementary Information:**

The online version contains supplementary material available at https://doi.org/10.1186/s13071-026-07569-w.

## Background

Zika virus (ZIKV), an arbovirus first isolated in Uganda, has undergone unprecedented global expansion over the past two decades [1]. While historically associated with mild, non-specific symptoms, epidemics in Micronesia (2007) [2], French Polynesia (2013–2014) [3], and the Americas (2015) revealed severe neurological complications, prompting international public health concern [4]. In Africa, ancestral ZIKV transmission is historically documented at largely asymptomatic or sporadic levels, although recent outbreaks in Cape Verde and Angola linked African transmission settings to congenital abnormalities and microcephaly. Genomic analyses demonstrated that these outbreaks were driven by Asian-lineage strains rather than local African lineages [5, 6]. Consequently, the contribution of ancestral African ZIKV lineages to viral emergence and transmission dynamics remains poorly understood, even as increasing regional and global connectivity may facilitate broader dissemination [7].

Despite the African origin of ZIKV, relatively little is known about interactions between Old-World African strains and Neotropical *Aedes aegypti* populations, the principal urban vector responsible for transmission in the Americas. Conversely, the susceptibility of African *Ae. aegypti* populations to New-world ZIKV strains remains similarly under characterized, though initial evidence suggests that African vectors may exhibit lower baseline susceptibility to the introduced American/Asian lineages [8, 9]. Vector competence is strongly influenced by mosquito immunity [10], notably the Toll, JAK–STAT [11–15], and RNA interference (RNAi) pathways [16]. However, arboviruses have evolved multiple strategies to evade or suppress these antiviral defenses, and the molecular mechanisms governing these interactions remain incompletely understood.

In addition to host immune responses, viral genetic variation plays a critical role in determining vector competence by influencing midgut infection, dissemination, immune evasion, and transmission efficiency [17]. Even closely related arboviral isolates can differ substantially in their ability to infect and be transmitted by mosquitoes [17]. Previous studies have shown that mutations in coding and non-coding viral regions can alter viral fitness, immune modulation, and adaptation across mosquito and vertebrate hosts [18]. For example, specific amino acid substitutions in West Nile virus have been associated with enhanced avian viremia and increased mosquito transmission efficiency, illustrating how viral evolution can shape epidemic potential [19].

Vector susceptibility is also influenced by intrinsic mosquito transcriptional profiles. For instance, *Ae. aegypti* populations refractory to dengue virus exhibit elevated basal immune activation compared with susceptible populations, suggesting that constitutive immune states may influence arboviral transmission [20]. Based on these observations, we hypothesized that Old-World ZIKV strains would induce stronger mosquito immune responses and lower viral replication, whereas Neotropical strains would exhibit enhanced replication and dissemination. To test this hypothesis, we compared the vector competence of Mexican *Ae. aegypti* mosquitoes for genetically distinct Old-World and Neotropical ZIKV strains and characterized strain-specific mosquito transcriptomic responses associated with viral transmission efficiency.

## Methods

### Viruses and mosquitoes

The ZIKV strains used in this study were obtained through BEI Resources, NIAID, NIH. Strain NR-50355 (ZIKV R103451; Human/2015/Honduras, GenBank accession no. KX262887) was isolated on January 6, 2016, from the placenta of a traveler returning from Honduras in 2015 [21]. Strain NR-50338 (ZIKV DAK AR 41524; Mosquito/1984/Senegal, GenBank accession no. KX601166) was isolated from *Ae. africanus* collected in Kédougou, Senegal, on November 17, 1984, and subsequently passaged three times at BEI Resources [22]. Viral isolates were acquired under Texas Tech University Institutional Review Board-approved protocols, with a material transfer agreement from the NIH outlining the permitted use and restrictions.

Mexican *Ae. aegypti* mosquitoes, originally collected from Poza Rica, Mexico, in 2016 and maintained to generation F35, were generously provided by A. Ciota (New York State Department of Health) and used in this study.

### Propagation of cells and viruses

Vero CCL-81 cells (ATCC) were freshly obtained and maintained in Dulbecco’s Modified Eagle Medium (DMEM; ThermoFisher Scientific, 11965092) supplemented with 10% fetal bovine serum (FBS; Gibco, 16140071) and 1% penicillin/streptomycin (Gibco, 15140148). Media were sterilized using a 0.2 μm filter unit (ThermoFisher Scientific, 567–0020). Cells were cultured in T25 flasks (5 ml per flask) and incubated at 37 °C with 5% CO₂, monitored daily for morphology and confluency using an EVOS XL Core microscope. Cultures were passaged when they reached ~ 90% confluency.

ZIKV stocks were amplified by three passages in Vero CCL-81 cells. Briefly, confluent monolayers were inoculated with 140 μl of viral suspension and incubated at 37 °C for 1 h, with gentle rocking every 15 min. Control flasks were mock-infected with an equal volume of maintenance media. After adsorption, 5 ml of DMEM supplemented with 10% FBS was added, and cultures were incubated at 37 °C with 5% CO₂ for 96 h [23]. Supernatants were harvested, clarified by centrifugation at 300 × g for 10 min at 4 °C, and stored at −  80 °C until plaque titration [16].

### Determination of virus titer by plaque assay

To determine viral titers, 3 ml of Vero CCL-81 cells (2.0 × 10^5^ cells/ml) were seeded into each well of a 6-well plate and maintained in DMEM supplemented with 10% FBS at 37 °C with 5% CO₂ until reaching ~ 90% confluency. Cell growth was monitored daily using an EVOS XL Core Imaging System. Confluent monolayers were inoculated with 100 μl of eightfold serially diluted ZIKV. Undiluted frozen ZIKV served as the positive control, while negative control wells were mock inoculated with DMEM. All samples were plated in duplicate [24].

Following infection, each well was overlaid with 3 ml of methylcellulose and incubated for 96 h at 37 °C with 5% CO₂ until plaques were visible under the microscope. On day 4, the overlay was aspirated, and cells were fixed overnight at room temperature with 3 ml of 10% formaldehyde and then stained overnight with 2 ml of 0.5% crystal violet.

Plaques were counted from wells containing 10–80 discrete plaques. Viral titers were calculated by multiplying the average plaque count from duplicate wells by the reciprocal of the dilution factor and adjusting for inoculum volume [25]. The integrity of both ZIKV isolates was confirmed by aligning RNAseq reads to the corresponding ZIKV reference genomes (GenBank accession nos. KX262887 and KX601166).

### Mosquitoes

The larvae of the F35 generation of *Ae. aegypti* eggs were maintained in plastic rectangular flat containers [35.6 cm length × 27.9 cm width × 8.3 cm height] (Sterilite, catalog no. 1963) at a density of 1 larva per 5 ml of dechlorinated water and reared at 60% relative humidity and a light–dark (LD) photoperiod of 14:10 h. The photoperiod was consistent for infected mosquitoes too. The *Ae. aegypti* eggs used in this study were molecularly identified by amplifying and sequencing the cytochrome oxidase I gene. The larvae were fed Tetra Pond Koi growth feed [26]. The adult male and female were transferred to cages and housed together for 8 days at 28 °C at a relative humidity of 60% while being provided with sugar and water ad libitum [26]. To stimulate blood feeding, female mosquitoes were starved of water and sugar for 5 h before an infectious blood meal according to our insectary laboratory standard operating procedures. The blood meal consisted of a 1:1 dilution of defibrinated sheep blood and Zika virus mixed to a final concentration of 2.5% sucrose solution. Infectious blood meals contained either 7.0 log_10_ PFU/ml Neotropical (ZIKV Honduras) or 7.0 log_10_ PFU/ml Old-World (ZIKV Dakar) strains. The infectious blood meal was delivered in a 37 °C pre-heated Hemotek membrane feeding system (Discovery Workshops, Accrington, UK) with a porcine sausage casing membrane. After an hour of feeding, the mosquitoes were anesthetized with CO_2_ and immobilized on a pre-chilled tray connected to 100% CO_2._ Engorged females were isolated and placed into individual plastic cylindrical containers (30 female individuals per cylinder). Three biological replicates of each ZIKV strain were included in the experiment. A total of *n* = 180 samples were exposed to an infectious blood meal in this study. Infected females were maintained on a 10% sucrose solution provided ad libitum and incubated at 28 °C at a relative humidity of 60% [26]. The blood meal was titered to exclude titer decay during membrane feeding.

### Vector competence analysis

Vector competence analysis was performed as previously described [27]. At 14 days post-infection (dpi), female mosquitoes were immobilized with triethylamine (Sigma-Aldrich, St. Louis, MO, USA). This 14-dpi time point was selected as a definitive endpoint to capture mature, late-phase infection and maximum transmission efficiency. This interval ensures sufficient time for viral replication to surpass anatomical bottlenecks, escape the midgut, and accumulate in the salivary glands [28, 29]. To assess ZIKV dissemination, legs were removed and placed in individual tubes for screening. Saliva was collected under sterile conditions by allowing mosquitoes to insert their proboscis into a capillary tube containing ~ 20 µl of filter-sterilized fetal bovine serum mixed 1:1 with 50% sucrose for 30 min, after which the mixture was expelled into 125 µl of filter-sterilized mosquito diluent [20% heat-inactivated fetal bovine serum in Dulbecco's phosphate-buffered saline supplemented with penicillin/streptomycin (50 µg/ml), gentamicin (50 µg/ml), and Fungizone (2 µg/ml); Sigma-Aldrich]. Transmission was determined by screening saliva samples for ZIKV. Whole bodies were collected in sterile microcentrifuge tubes to estimate infection rates. All samples (legs, saliva, bodies) were stored at –80 °C until analysis.

Absolute viral copy numbers were quantified using a ZIKV-specific real-time RT-qPCR assay targeting the NS1 region [30].

### Assessment of strain-specific vector competence

To assess the vector competence of Mexican *Ae. aegypti* for both strains of ZIKV, a total of 152 mosquitoes were screened; a total of 28 mosquitoes died during the incubation stage. RNA was extracted from mosquitoes using QIAamp viral RNA kit (Qiagen, 52,906) according to the manufacturer's protocol. Briefly, a single 2 mm Zirconia silicate bead was added to a tube containing the whole body or leg. The whole body or legs were homogenized in a Mini-BeadBeater-16 (BioSpec products USA), set at a frequency of 3450 Hz for two minutes. The supernatants of the homogenates were lysed with lysis buffer. In the case of saliva samples, the lysis buffer was added directly to the saliva. Carrier RNA was added to the lysis buffer to enhance the binding of viral RNA to the columns. The lysate was incubated at room temperature to ensure complete lysis. To concentrate the RNA, an equal volume of absolute ethanol was added to the lysed tissues, and the solution was loaded onto a spin column to bind the RNA to the column. The column-bound RNA was washed twice with wash buffer to remove contaminants and stabilize the RNA. Finally, the RNA was eluted into a clean microcentrifuge tube. The RNA was stored at −80 °C until downstream procedures.

### Vector competence metrics and tissue analyses

To rigorously quantify vector competence, individual mosquito tissues were harvested at 14 dpi and evaluated using three standard epidemiological metrics. Infection rate was calculated as the proportion of blood-fed mosquitoes with ZIKV-positive bodies out of the total number of blood-fed mosquitoes tested. Dissemination rate was determined by calculating the proportion of infected mosquitoes that also possessed ZIKV-positive secondary tissues, specifically utilizing the legs to evaluate the capacity of the virus to bypass the midgut barrier and spread systematically. Finally, transmission efficiency was defined as the proportion of disseminated mosquitoes (those with ZIKV-positive legs) that yielded ZIKV-positive saliva, measuring the ultimate capability of the virus to invade the salivary gland and secrete infectious particles into the expectorate.

### Zika virus RNA quantification by quantitative real-time PCR

To quantify copies of the ZIKV genome in RNA samples, 5 μl of total RNA was reverse transcribed using the qScript 1-Step Virus Tough Mix (Quantabio) on a thermocycler (BioRad CFX96 Real-Time System). Reverse transcription was carried out at 55 °C for 10 min, followed by inactivation of the cDNA synthesis step at 95 °C for 1 min.

Amplification of the ZIKV NS1 gene was performed using the following thermal cycling conditions: 95 °C for 10 s and 60 °C for 60 s, repeated for 45 cycles. Primers and probes targeting the NS1 region were selected due to its high conservation across ZIKV strains (approximately 99.3% sequence identity), ensuring efficient and reproducible amplification. The primers used in this study were: ZIKV 1086 CCGCTGCCCAACACAAG; ZIKV 1162 C CCACTAACGTTCTTTTGCAGACAT; ZIKA 1107-FAM AGCCTACCTTGACAAGCAGTCAGACACTCAA) [31]. Positive control consisted of frozen ZIKV, while sterile water served as the negative control. A non-template control (NTC) was also included in each run to monitor for contamination. To determine the viral genome, copy number, a standard curve was constructed using an eightfold serial dilution of plaque-titered ZIKV stock. Viral load differences were statistically analyzed using one-way ANOVA in GraphPad Prism 10. A threshold of 2.0 log_10_ ZIKV genome copies was set as the limit of detection [32], and any sample above this value was considered positive. Significant differences in viral titers were observed across tissues.

To validate RT-qPCR findings, plaque assays were conducted on ten randomly selected samples per replicate from legs, saliva, and whole-body tissues. These samples were representative of those collected from each biological replicate and processed following the previously described plaque assay protocol.

### Assessment for strain-specific variation in gene expression profiles

Whole bodies confirmed to be infected were subjected to Illumina transcriptome sequencing. A total of 18 *Ae. aegypti* either orally exposed to a noninfectious blood meal, the Old-World ZIKV strain, or the Neotropical ZIKV strain (*N* = 6 per treatment, 7.0 log_10_ PFU/ml for each infectious blood meal) (Supplementary file 1). Total RNA was isolated from individual mosquitoes that had been orally exposed to one of three different blood meal types [Non-infectious (NINF) blood meal, *n* = 6; ZIKV Neotropical blood meal, *n* = 6; and Old-World blood meal, *n* = 6]. RNA extraction was performed as previously described. ZIKV infection status was confirmed in both whole bodies and corresponding legs using RT-qPCR, as described above. Mosquitoes in which both the body and legs tested positive for ZIKV were identified for RNAseq analysis.

Following RNA extraction, total RNA was treated with DNase using the Ambion TURBO DNA-free kit (ThermoFisher Scientific) to eliminate genomic DNA contamination. RNA quality and integrity were assessed using the Agilent 2100 Bioanalyzer with the RNA 6000 Nano Chip. mRNA libraries were then prepared using the Illumina NEBNext Prep Kit (New England Biolabs), according to the manufacturer’s protocol. All 18 libraries were pair-end sequenced (250 bp reads) on an Illumina MiSeq system (500-cycle kit) at the Centre for Biotechnology and Genomics at Texas Tech University (CBGTTU). Samples from all experimental groups were evenly distributed across sequencing lanes, minimizing confounding between biological condition and lane effects. Raw sequencing data have been deposited in the NCBI Bio Project database under accession number PRJNA1314310.

Gene expression levels were analyzed using CLC Genomics Workbench 12 (QIAGEN). Sequencing generated raw reads in FASTQ format, and to increase coverage, paired-end files from different sequencing lanes were concatenated into single files per sample. These files were then trimmed and filtered to remove ambiguous nucleotides and low-quality reads.

As part of quality control, raw data were evaluated for yield, error rate and Phred quality scores (Q-scores). Yield refers to the amount of usable data and the depth coverage per gene, which enhances the accuracy of expression comparisons between treatment groups [33]. Error rate indicates the frequency of base call mismatches, which may affect the accuracy of gene expression quantification [34]. Phred scores reflect the probability of base call errors, with higher scores indicating greater confidence [35].

High-quality reads were mapped to the *Ae. aegypti* reference genome AaegL5.0 (GenBank accession no. GCA_002204515.1) using CLC’s core read mapping algorithm. Reads aligning uniquely to transcripts were quantified to assess expression levels.

Differential expression (DE) was calculated using DESeq2 [36] within CLC Genomics Workbench. DESeq2 performs both normalization and DE analysis, correcting for variation in sequencing depth and library size. Significance of differential expression was assessed using an exact test for the negative binomial distribution, with false Discovery rate (FDR) correction at *P* < 0.05.

Functionally enriched genes were annotated by BLASTx against public databases, using an E-value cutoff of 1e-10 and a bit score threshold of 200. To understand the biological significance of the differentially expressed genes (DEGs) identified in the above RNAseq data, Gene Ontology (GO) analysis was performed within CLC Genomics Workbench. Enrichment of Gene Ontology (GO) terms was performed using AmiGO [37]. GO analysis was used to classify genes into three functional domains: molecular function, biological process, and cellular component [38], enabling interpretation of functional patterns across treatments.

### ZIKV whole-genome sequencing and viral stock integrity verification

We performed full ZIKV genome sequencing to ensure viral stock integrity. Viral RNA was extracted from infected cell culture supernatants using the QIAamp Viral RNA Mini Kit according to the manufacturer’s instructions. Complementary DNA (cDNA) was synthesized using random hexamer priming, followed by Illumina-compatible library preparation for paired-end sequencing at CBGTTU. Libraries were sequenced on an Illumina MiSeq platform (500-cycle kit), generating 2 × 150 bp reads. Raw FASTQ files underwent quality control and adapter trimming using fastp (v1.1.0) with automatic paired-end adapter detection, and low-quality bases and short reads were removed prior to downstream analysis. Both genomic sequences have been submitted to NCBI under the accession number PRJNA1426502.

### Read mapping and variant calling

Trimmed reads were aligned to lineage-appropriate Zika virus reference genomes using BWA-MEM (v0.7.19). Specifically, the Neotropical isolate was mapped to the ZIKV Honduras 2015 reference genome (GenBank: KX262887), while the Old-World isolate was aligned to the ZIKV Senegal 1984 reference genome (GenBank: KX601166). Resulting alignments were sorted and indexed using samtools to facilitate downstream analyses. Variant calling was performed using bcftools mpileup followed by bcftools call, and genome-wide coverage statistics were generated with samtools coverage to confirm uniform sequencing depth across coding regions. Variants were filtered to retain high-confidence calls using the following criteria: minimum total read depth (DP ≥ 10), minimum alternate allele depth ≥ 10, and alternate allele frequency ≥ 75%. Filtered variants were subsequently incorporated into consensus genome sequences using bcftools consensus.

### Functional annotation and protein mapping

Functional annotation of variants was performed using SnpEff (v5.4a) with custom databases constructed from GenBank reference annotations. Amino acid substitutions were extracted from the ANN field according to SnpEff standard format.

To assign coding variants to individual viral proteins, polyprotein cleavage boundaries were derived from annotated mat_peptide features in the GenBank reference KX601166. Amino acid positions were mapped to mature protein regions (capsid, pr, M, E, NS1–NS5) based on annotated cleavage coordinates. Custom AWK scripts were used to generate structured tables summarizing genomic position, amino acid position, protein assignment, variant classification (synonymous or missense), genotype, and read depth metrics.

To ensure coordinate consistency, the Old-World isolate was re-aligned to the ZIKV Honduras 2015 reference genome (KX262887) using BWA-MEM, and variants were re-called with bcftools mpileup and call under identical parameters. Normalized VCF files were compared using bcftools isec to identify isolate-specific and shared substitutions. To assess functional divergence, variants were annotated using SnpEff against the ZIKV Honduras 2015 reference genome (KX262887). Of 1177 annotated nucleotide substitutions distinguishing the Old-World and Neotropical isolates, 1040 (88.4%) were synonymous, 116 (9.9%) were nonsynonymous (missense), and no stop-gained mutations were detected. Thus, despite approximately 10.9% nucleotide divergence across the ~ 10.8 kb coding genome, the majority of substitutions do not alter encoded amino acids. Both isolates maintain an intact polyprotein without truncating mutations.

### Secondary blood feeding and fecundity assay

To evaluate whether transcriptional differences were reflected in phenotypic reproductive outcomes, females previously exposed to either NINF or ZIKV-infected blood (Old-World or Neotropical strains; described above) were offered a secondary noninfectious blood meal following the incubation period. Fully engorged females from the primary blood meal were maintained in replicate cages, each serving as an independent biological unit. During the secondary feeding, the number of females that successfully engorged (Fed) and those that did not feed (Nonfed) were recorded per cage. The oviposition cups were added to the cages 48 h after blood meal digestion. Following oviposition, total egg production per cage was quantified.

Feeding probability was analyzed using a binomial generalized linear mixed model (GLMM) with logit link, modeling the probability of feeding (Fed versus Nonfed females) as a function of Treatment and including Cage as a random intercept to account for biological replication. To assess fecundity, total egg counts at the cage level were modeled using a Poisson GLMM with log link. To account for variations in the number of responding mosquitoes across cages, we incorporated log (Fed) as an offset. This standard approach models the cage-level egg production rate per fed female without assuming uniform individual reproductive output or that females successfully laid eggs, while strictly preserving the cage as the primary unit of analysis. Cage was included as a random intercept. Model fit was evaluated using Pearson residual-based dispersion diagnostics, which indicated no evidence of overdispersion. All analyses were conducted in R using the lme4 package, with significance assessed at α = 0.05.

## Results

### Vector competence of Mexican *Aedes aegypti* for Neotropical and old world ZIKV strains

The *Ae. aegypti* Mexico population showed similar susceptibility to both ZIKV strains, with infection rates of 70% (60/86) for the Neotropical ZIKV strain and 57% (45/81) for the Old-World ZIKV strain (Fisher’s exact test, *P* = 0.776) (Supplementary file 2, Fig. 1A). However, dissemination and transmission rates were significantly higher for Neotropical ZIKV strain. A total of 90% (54/60) of mosquitoes infected with the Neotropical ZIKV strain exhibited viral dissemination compared with only 7% (3/45) for Old-World ZIKV strain (*P* < 0.0001) (Supplementary file 2, Fig. 1B). Likewise, transmission rates reached 96% (52/54) for Neotropical ZIKV strain, while only 33% (1/3) of Old-World ZIKV strain infected mosquitoes transmitted the virus (*P* < 0.0001) (Supplementary file 2, Fig. 1C).

**Fig. 1:**
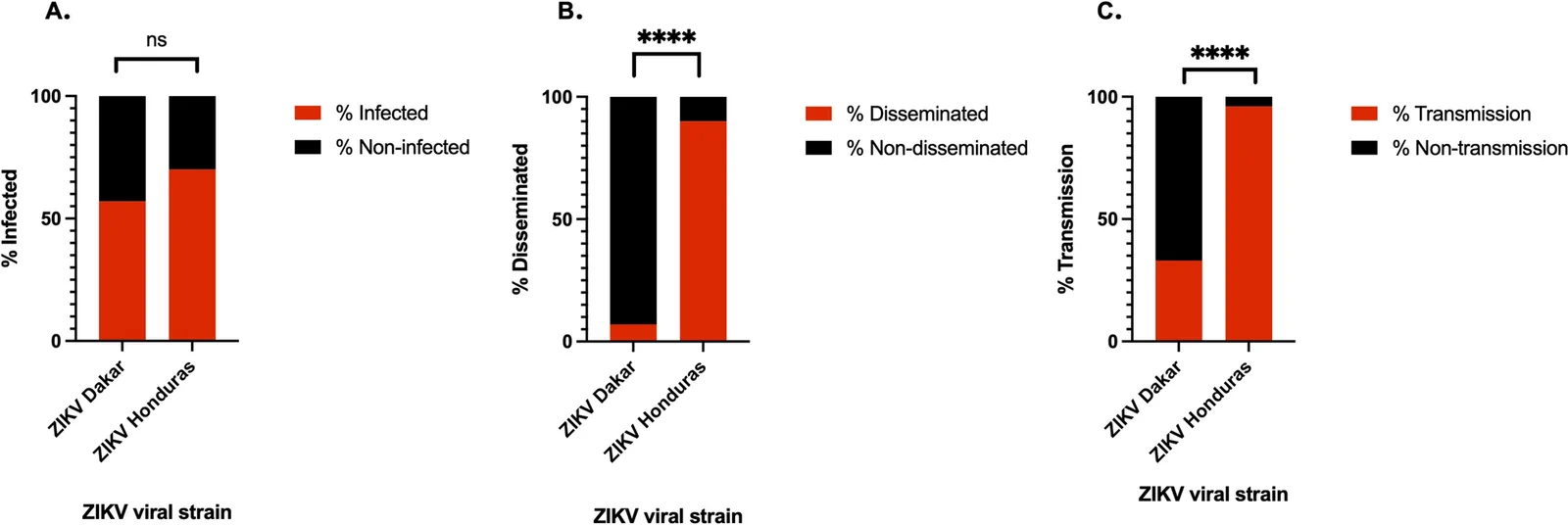
Vector competence of Mexican *Aedes aegypti* mosquitoes for Old-World and Neotropical ZIKV strains. **A** Whole-body infection rates, **B** dissemination rates, and **C** transmission rates following oral exposure to infectious blood meals containing either Old-World or Neotropical ZIKV strains. Red bars represent the proportion of mosquitoes whose whole bodies were infected, disseminated, or transmitting, whereas black bars represent the total number of mosquitoes screened in each category. Numbers shown at the top of each bar indicate the number of positive individuals (red) over the total number screened (black). Statistical significance was determined using Fisher’s exact test. **P < 0.01; ****P < 0.0001

Whole bodies of mosquitoes infected with the Neotropical ZIKV strain harbored significantly higher viral genome copies than those exposed to the Old-World strain (unpaired *t* test, *P* = 0.0003; *t* = 3.708, df = 150), indicating that while Mexican *Ae. aegypti* were equally susceptible to both strains of ZIKV, the Neotropical strain replicated more efficiently (Supplementary file 2, Fig. 2A). Likewise, disseminated viral RNA copy numbers were markedly higher for Neotropical ZIKV strain (*P* < 0.0001; *t* = 6, df = 91), suggesting that Old-World ZIKV strain encounters strong barriers to systemic dissemination, whereas Neotropical ZIKV strain overcomes these barriers effectively (Supplementary file 2, Fig. 2B). Finally, mosquitoes infected with Neotropical ZIKV strain transmitted substantially higher viral genome copies in saliva compared to Old-World ZIKV strain (*P* < 0.0001; *t* = 17.67, df = 54), demonstrating the superior transmission potential of the Neotropical strain (Supplementary file 2, Fig. 2C).

**Fig. 2:**
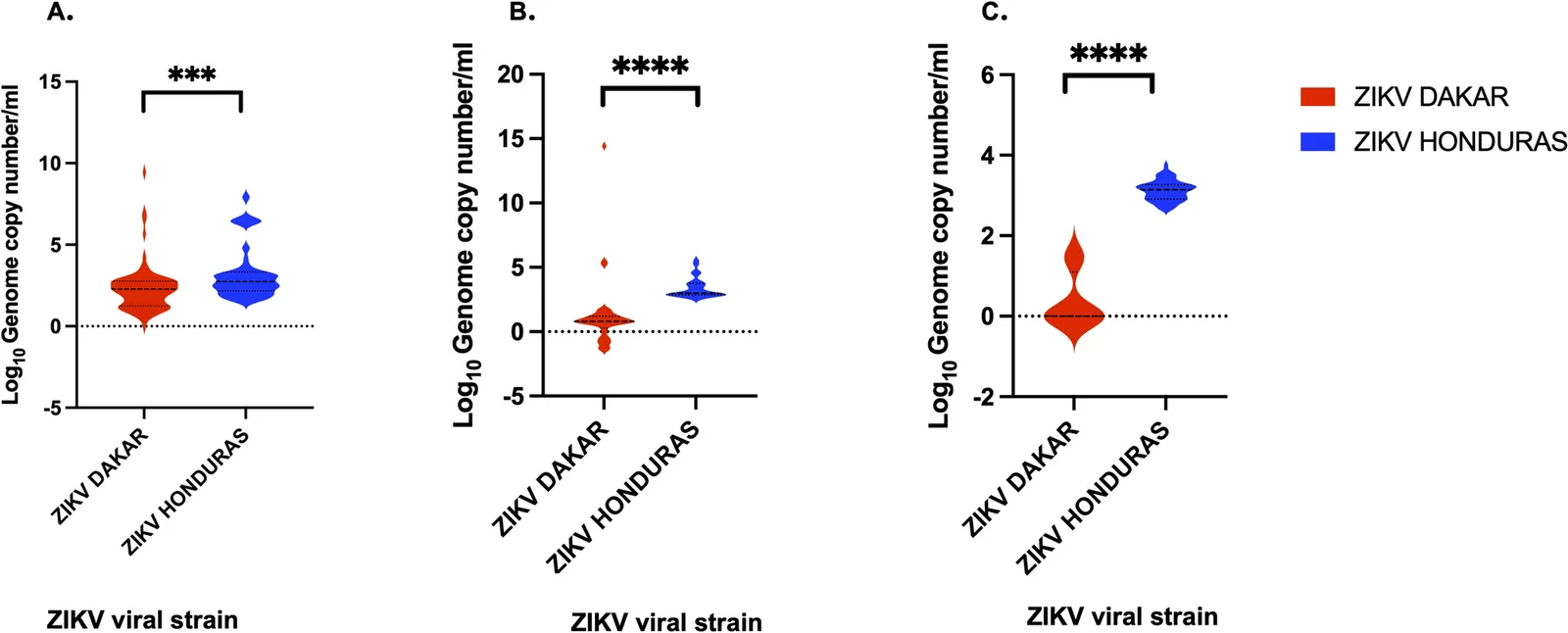
Viral load of ZIKV in *Aedes aegypti* Mexico populations. The box violin plot represents the absolute viral load of ZIKV in individuals exposed to different strains of ZIKV (Neotropical and Old-World). The central line within the box represents the median value of the dataset. The length of the box represents the interquartile range, the range between the 25th and 75th percentiles. The length of the lines extending from the box represents the range of the data. Datapoints extending beyond the whiskers are outliers. The width of the violin at a particular point represents the density of the data points clustering at a point. Statistical significance is indicated by * (*P* < 0.05), ** (*P* < 0.01) and *** (*P *< 0.001); **A** viral load of infected bodies, **B** viral loads of disseminated virus, and **C** viral loads of transmitted virus

### Sequencing output and data quality assessment

After trimming and filtering, ~ 1.66 billion high-quality reads were retained across all samples, with an average of ~ 92 million reads per individual (range: 52–136 million). Mapping efficiency was consistently high, with 62–92% of reads aligning to annotated genes. Poly(A) selection during library preparation resulted in effective enrichment of mRNA, with 85–94% of reads mapping to annotated transcripts and only 3–12% corresponding to residual rRNA (Supplementary File 3).

### Core and strain-specific gene regulation during ZIKV infection

**Fig. 3:**
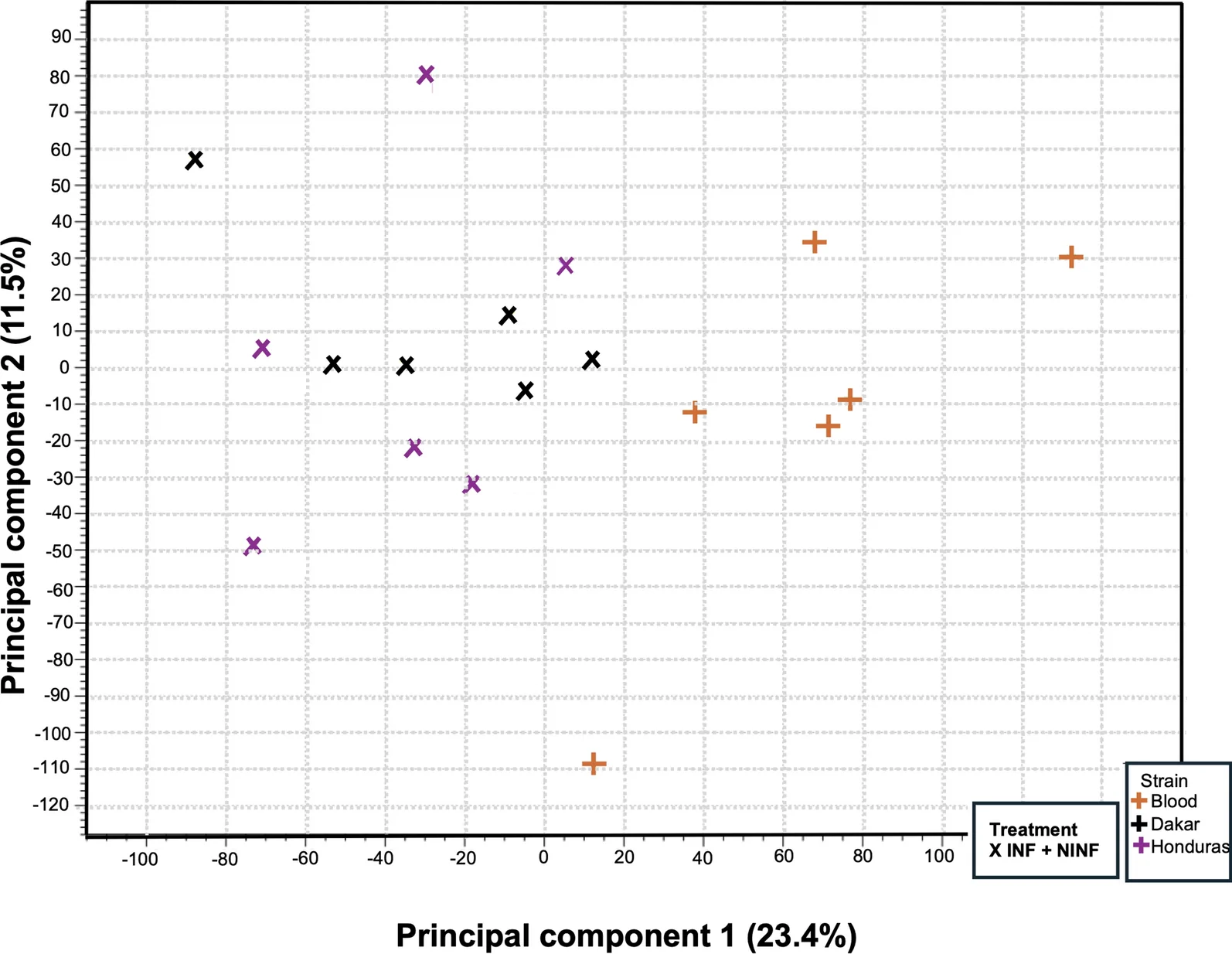
Principal Coordinates Analysis (PCA) of transcriptomic profiles from *Aedes aegypti* mosquitoes exposed to noninfectious blood meals (Blood) or infectious blood meals containing Old-World (Dakar) or Neotropical (Honduras) strains. Each point represents an individual mosquito sample, and clustering reflects similarities in global gene expression profiles among treatments. PC1 and PC2 represent the first two principal components and explain 23.4% and 11.5% of the total variance, respectively

Principal component analysis (PCA) of the transcriptomic profiles revealed distinct clustering patterns based on blood meal type. Mosquitoes fed on noninfectious blood meals formed a clearly separated cluster, underscoring a unique gene expression signature relative to those exposed to ZIKV. In contrast, mosquitoes infected with either the Neotropical or Old-World ZIKV strains displayed overlapping but partially distinct transcriptional responses (Fig. 3). Notably, individual samples, such as Hond6 and Hond2, showed greater dispersion within the Neotropical group, suggesting intra-strain variability in transcriptional responses among mosquitoes exposed to the same viral strain.

**Fig. 4:**
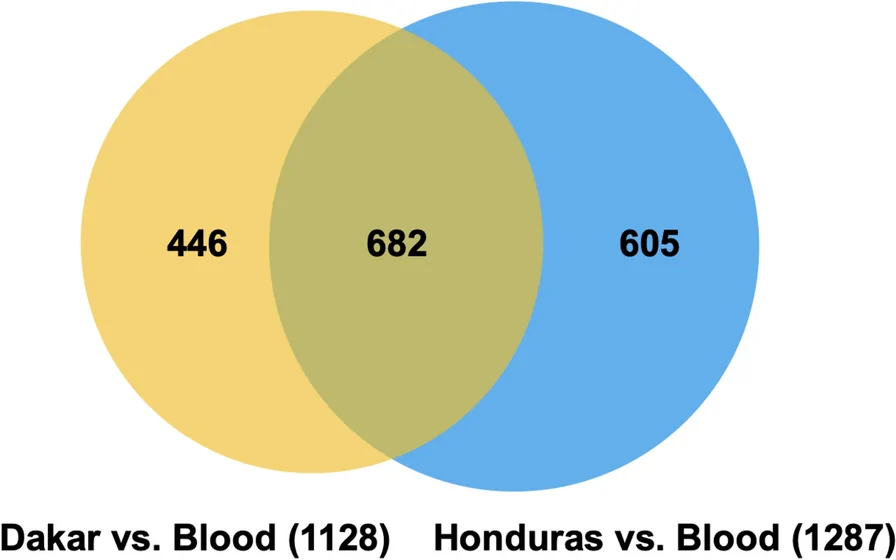
Venn diagram showing the overlap of differentially expressed genes between Old-World ZIKV strain (yellow) and Neotropical ZIKV strain (blue). A total of 446 genes were unique Old-World ZIKV strain, and 605 genes were unique to Neotropical ZIKV strain, while 682 genes were shared between the two groups

Differential expression analysis revealed that 1128 genes were altered in response to the Old-World ZIKV strain, while 1287 genes were differentially expressed following infection with the Neotropical ZIKV strain. Of these, 682 DEGs were commonly regulated in both infection groups compared to blood-fed controls, whereas 446 were unique to Old-World ZIKV strains and 605 unique to Neotropical ZIKV strain. This indicates the presence of a conserved core transcriptional program during ZIKV infection, alongside strain-specific gene regulation that may reflect differences in viral replication dynamics and mosquito–virus interactions (Fig. 4).

### ZIKV infection reproductive trade-offs in Mexican *Aedes aegypti*

**Fig. 5:**
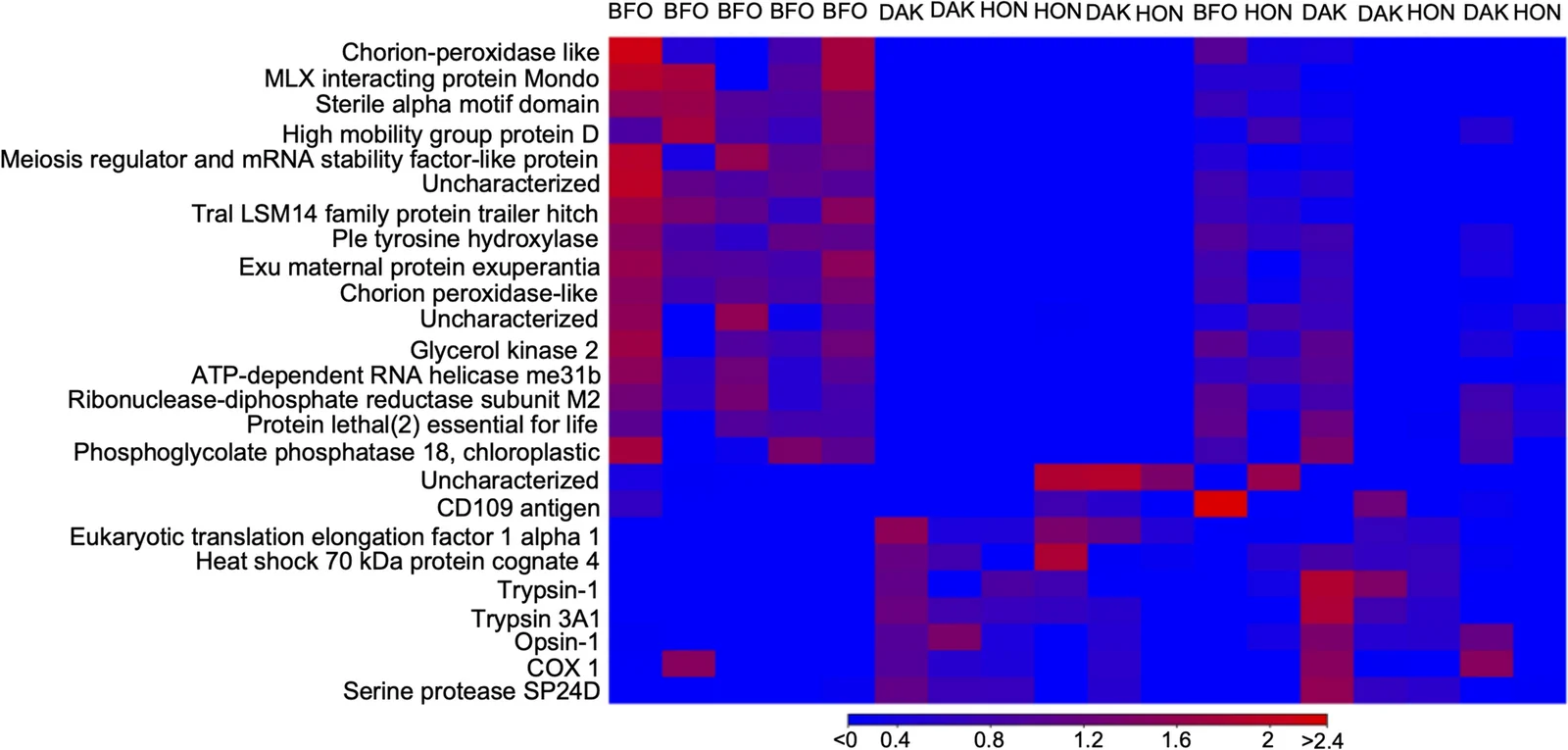
Heatmap of differentially expressed genes in mosquitoes exposed to different viral strains. Rows represent individual genes, and columns represent biological replicates. The color scale indicates relative expression levels, with red representing higher expression and blue representing lower expression

Heat map results further demonstrated that the chorion peroxidase-like gene, associated with eggshell formation, was upregulated only in mosquitoes fed on noninfectious blood meals, but not in either ZIKV-infected group. Additionally, several genes involved in metabolic and signaling pathways were more strongly upregulated in mosquitoes exposed to noninfectious blood meals compared to those infected with ZIKV (Fig. 5). Post hoc comparisons (Tukey adjusted) confirmed that ZIKV strains significantly reduced egg production relative to non-infected controls (p = 0.011 for Old-World; *P *< 0.0001 for Neotropical). Although the Neotropical strain exhibited a larger reduction in fecundity compared to Old-World, this difference did not reach statistical significance (*P* = 0.078).

### Distinct transcriptomic responses to old-World and neotropical ZIKV strains in Mexican *Aedes aegypti*

**Fig. 6:**
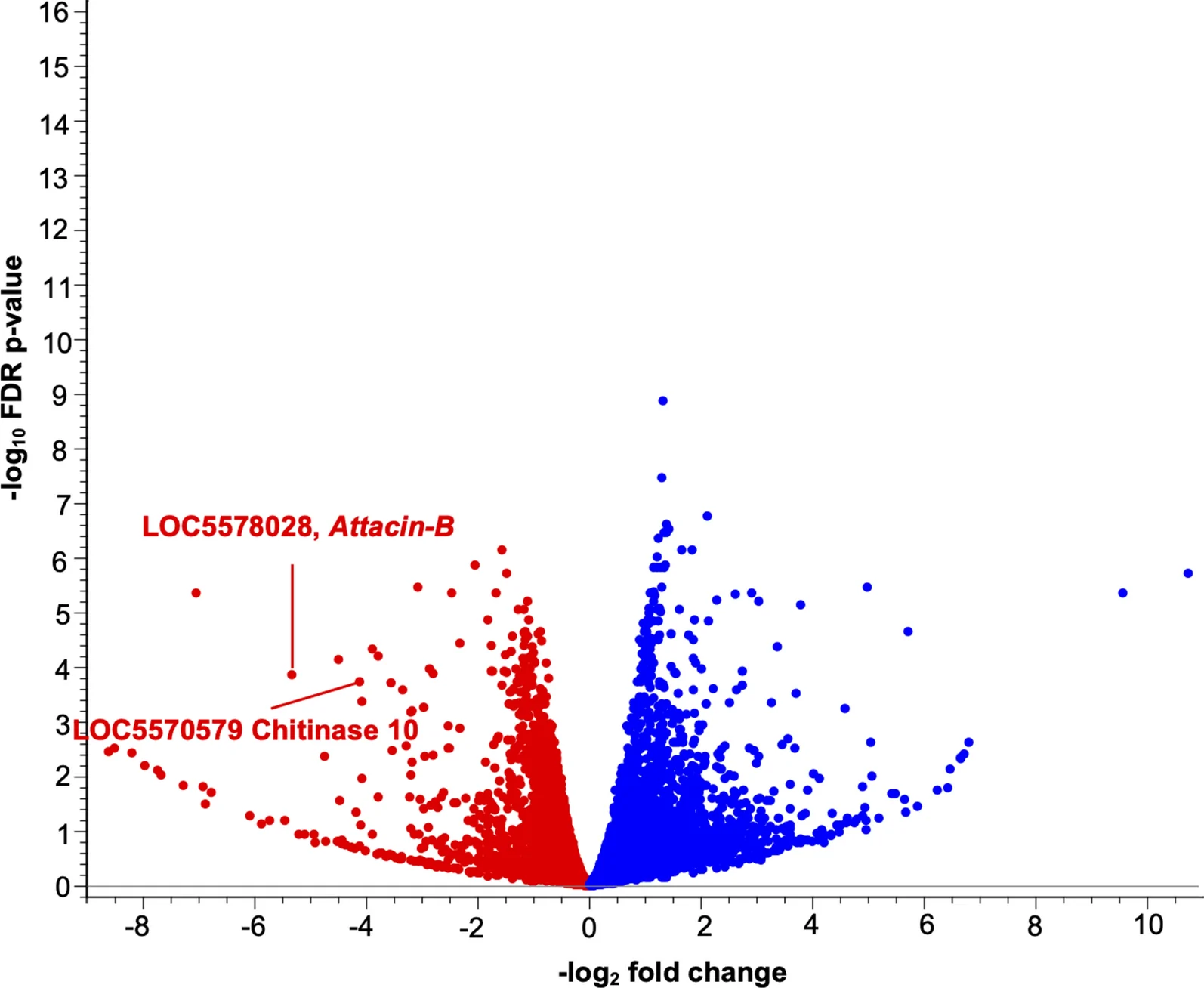
Volcano plot of differentially expressed genes in virus-infected mosquitoes. The x-axis represents the log_2_-fold change in gene expression, and the y-axis represents the − log_10_ adjusted *P* value. Red dots indicate significantly downregulated genes, while blue dots indicate significantly upregulated genes

A total of 19,241 genes were analyzed for differential expression. Of these, 1320 genes were differentially expressed in mosquitoes fed on Old-World ZIKV-infected blood meal compared to a noninfectious control (FDR P ≤ 0.05). Among them, 50 genes were significantly upregulated (log_2_-fold change ≥ 2; FDR P ≤ 0.05) and 42 were downregulated (log_2_-fold change ≤ − 2; FDR P ≤ 0.05) (Supplementary File 4). In contrast, infection with the Neotropical ZIKV strain resulted in 1437 differentially expressed genes relative to controls, including 99 upregulated and 55 downregulated (Supplementary File 5). Notably, infection with the Neotropical strain suppressed genes involved in immune response (LOC5578028, *Attacin-B*) and peritrophic matrix remodeling (LOC5570579, *Chitinase-10*) (Supplementary File 5; Fig. 6).

GO enrichment analyses of cellular component and molecular function for both strains revealed significant reliance on host translation machinery, with enrichment of ribosomes, ribosomal subunit, and ribonucleoprotein complexes-related terms (Supplementary File 6, 7).

Biological process GO terms further emphasized translation and protein biosynthesis across both strains, including cytoplasmic translation, ribosomal subunit assembly, ribonucleoprotein complex assembly, and peptide biosynthesis (Supplementary File 6, 7). In mosquitoes exposed to Old-World ZIKV, additional enrichment of protein refolding and anion transmembrane transport was observed, consistent with stabilization of viral/host proteins and enhanced viral entry/egress (Supplementary File 6). In contrast, Neotropical ZIKV infection was associated with GO terms linked to DNA replication and repair, consistent with host cell-cycle and DNA damage responses, as well as immune and defense processes (Supplementary File 7).

### Genome recovery and coding variation across the ZIKV polyprotein

Near-complete Zika virus genomes were successfully recovered for both isolates, with genome coverage exceeding 98% in each case. The Neotropical isolate achieved 99.89% genome coverage with a mean sequencing depth of 3245 ×, reflecting a deep coverage across the viral genome. Four nucleotide substitutions were identified relative to the ZIKV Honduras 2015 reference genome (GenBank: KX262887) at positions 2304 (A → G), 3012 (A → C), 4214 (G → A), and 7618 (C → T), each supported by high read depth (> 200 ×) and fixed within the consensus sequence (genotype 1/1).

**Fig. 7:**
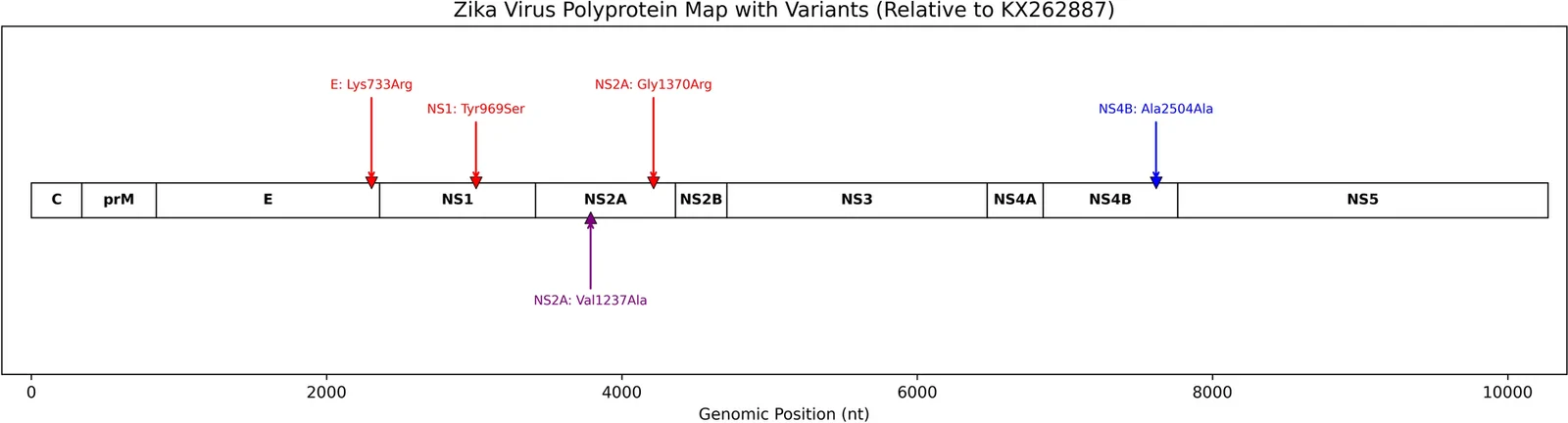
Genome coverage and coding variation in Neotropical and Old-World ZIKV isolates. Near-complete genomes (> 98% coverage) were recovered for both isolates. The Neotropical strain achieved 99.89% coverage (mean depth 3245 ×) and harbored four fixed nucleotide substitutions, three of which were nonsynonymous (E: Lys733Arg; NS1: Tyr969Ser; NS2A: Gly1370Arg) and one synonymous (NS4B: Ala2504Ala), with no changes detected in C, prM, NS2B, NS3, NS4A, or NS5. The Old-World isolate achieved 98.95% coverage (mean depth ~ 43 ×) and retained a single high-confidence missense variant (Val1237Ala) in NS2A after stringent filtering. Variants were distributed across structural and nonstructural proteins but did not involve the viral polymerase

Three of these substitutions resulted in nonsynonymous amino acid changes distributed across both structural and nonstructural proteins. A missense mutation was detected in the envelope (E) glycoprotein (Lys733Arg), along with additional nonsynonymous substitutions in NS1 (Tyr969Ser) and NS2A (Gly1370Arg). The remaining substitution was synonymous and occurred in NS4B (Ala2504Ala). No coding substitutions were observed in the capsid (C), pr, M, NS2B, NS3, NS4A, or NS5 (RNA-dependent RNA polymerase) regions. Overall, substitutions were present in both structural and replication-associated proteins but did not involve the viral polymerase (Fig. 7).

The Old-World isolate achieved 98.95% genome coverage with a mean depth of approximately 43X. Two candidate single-nucleotide variants were initially identified; however, after applying stringent filtering criteria, only one high-confidence variant remained at genomic position 3788 (T → C). This substitution was supported by 19 total reads, with 12 reads supporting the alternate allele (alternate allele frequency ~ 80%). Functional annotation classified this mutation as a missense variant predicted to result in a Valine-to-Alanine substitution (Val1237Ala) within the viral polyprotein, corresponding to the NS2A coding region (Fig. 7).

No insertions, deletions, or structural rearrangements were detected in either genome. Collectively, these results indicate limited coding divergence relative to lineage-appropriate reference genomes, with substitutions primarily affecting envelope and replication-associated proteins while preserving the conserved polymerase region.

To directly quantify genomic differences between isolates, both consensus genomes were aligned to a common reference (ZIKV Honduras 2015; KX262887), and variants were re-called under identical parameters. Pairwise comparison identified 1179 nucleotide differences between the Neotropical and Old-World isolates. Relative to the Honduras reference backbone, the Dakar isolate harbored 1,176 isolate-specific substitutions, whereas the Neotropical isolate differed at only three positions; one variant was shared. Across the ~ 10.8 kb coding genome, this corresponds to approximately 10.9% nucleotide divergence, consistent with established genetic separation between African and Asian ZIKV lineages.

### Strain-specific effects of ZIKV infection on secondary blood feeding and fecundity

A binomial generalized linear mixed model (GLMM; logit link, cage as a random intercept) revealed no significant effect of treatment on the probability of feeding during the secondary blood meal (likelihood ratio test: χ^2^ = 1.35, *P* = 0.51), indicating that prior infection did not alter host-seeking or blood-feeding success.

**Fig. 8:**
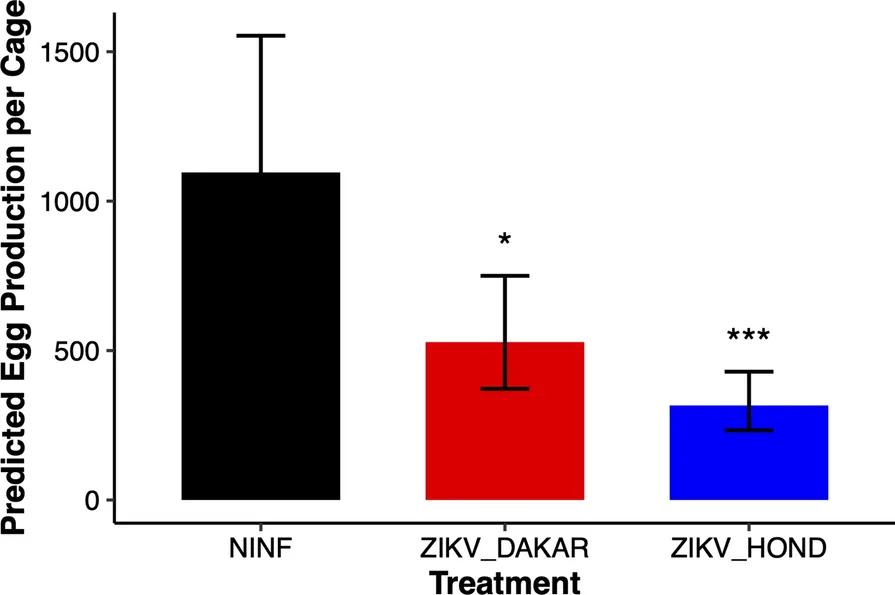
Strain-specific effects of ZIKV infection on fecundity in *Aedes aegypti* mosquitoes. Predicted egg production per cage following secondary blood feeding in mosquitoes previously exposed to noninfectious blood meals (NINF), Old-World ZIKV strain (ZIKV_DAKAR), or Neotropical ZIKV strain (ZIKV_HOND). Error bars represent standard error of the mean (SEM). Statistical significance is indicated as follows: *P < 0.05; ***P < 0.001

In contrast, egg production differed significantly among treatments. Fecundity was analyzed using a Poisson GLMM with a log link, incorporating log (Fed) as an offset to model egg production per fed female and including cage as a random effect. This model revealed a strong treatment effect on fecundity. Non-infected females produced an estimated ~ 111 eggs per fed female. In comparison, Old-World ZIKV-infected females produced approximately 35 eggs per female (β =  − 1.15 ± 0.59 SE, *P* = 0.051), representing an estimated 68% reduction relative to controls. Neotropical ZIKV-infected females produced approximately 18 eggs per female (β =  − 1.84 ± 0.55 SE, *P* < 0.001), corresponding to an estimated 84% reduction in fecundity (Fig. 8) (Supplementary File 8). Together, these results demonstrate substantial and strain-specific reproductive costs of infection, with the Neotropical strain imposing a markedly stronger fecundity suppression than the Old-World strain.

## Discussion

In this study, Mexican *Ae. aegypti* mosquitoes exhibited marked strain-associated differences in ZIKV dissemination and transmission. The Neotropical strain disseminated more efficiently, reached higher systemic viral loads, and was more frequently detected in saliva. In contrast, the Old-World isolate had a reduced compatibility with this mosquito population.

Genomic analysis identified nonsynonymous substitutions in the Envelope (E), NS1, and NS2A proteins of the Neotropical isolate that may contribute to enhanced vector competence. Mutations in these proteins have previously been associated with viral entry efficiency, immune evasion, replication, and transmission in flaviviruses [39, 40]. In particular, NS1 mutations in epidemic Asian-lineage ZIKV strains have been linked to increased mosquito infectivity and immune modulation [39, 40]. Collectively, these substitutions co-occurred with increased viral RNA accumulation and tissue dissemination observed in the Neotropical strain, highlighting them as candidate determinants of enhanced transmission efficiency.

In contrast, the Old-World isolate harbored only a single NS2A substitution and failed to disseminate successfully in this mosquito background. These findings support the possibility that strain-specific viral genetic variation contributes to differential compatibility with Neotropical *Ae. aegypti* populations. However, because only one representative isolate from each lineage was examined, the observed differences should be interpreted as strain-specific rather than universally lineage-wide.

Transcriptomic analyses further revealed strain-associated differences in mosquito gene expression profiles. Mosquitoes infected with the Neotropical strain exhibited reduced expression of immune- and barrier-associated genes, including *Attacin-B* and *Chitinase-10*. Chitinases contribute to peritrophic matrix remodeling and midgut barrier maintenance [41, 42], suggesting that altered expression of these genes may reflect changes in midgut physiology during infection. Similarly, reduced antimicrobial peptide expression has previously been reported during ZIKV infection in *Ae. aegypti* [43]*.* Although our study does not establish direct functional causality, these findings suggest that strain-specific modulation of mosquito immune and barrier responses may contribute to differences in dissemination and transmission efficiency.

Both ZIKV strains were additionally associated with enrichment of ribosome- and ribonucleoprotein-related gene ontology categories, consistent with the reliance of RNA viruses on host translational machinery [44]. These transcriptional signatures likely reflect altered cellular states during infection and increased demand for protein synthesis.

We also observed evidence of infection-associated reproductive costs. While ZIKV infection did not alter secondary blood-feeding success, infected mosquitoes produced substantially fewer eggs, particularly following exposure to the Neotropical strain. These findings were accompanied by reduced expression of a chorion peroxidase-like gene associated with eggshell formation [45]. Similar reductions in mosquito fecundity following arboviral or *Plasmodium* infection have been previously reported and are thought to reflect energetic trade-offs between reproduction and infection [46–48, 50]*.*

Several limitations should be considered when interpreting these findings. First, only a single laboratory-maintained *Ae. aegypti* population and one representative isolate from each lineage were examined and the midgut infection levels were not assessed. Second, the low dissemination efficiency of the Old-World strain limited the number of saliva-positive mosquitoes available for downstream transmission analyses. Third, transcriptomic findings were not independently validated using RT-qPCR. Finally, sequencing depth varied between isolates which may affect variant detection and comparative genomic interpretation. Future studies incorporating multiple mosquito populations, midgut viral loads, additional viral isolates, and functional validation approaches will be important to determine the broader generalizability and mechanistic basis of these observations.

Overall, our findings demonstrate that genetically distinct ZIKV strains can exhibit substantially different interactions with Neotropical *Ae. aegypti* mosquitoes. The enhanced dissemination and transmission efficiency observed for the Neotropical strain, together with associated genomic and transcriptomic differences, highlights viral genetic background as an important determinant of vector competence and outbreak potential.

## Conclusions

Our study identified amino acid variations in the Envelope (E), NS1, and NS2A proteins of the Neotropical ZIKV strain that were associated with enhanced viral dissemination in Mexican *Ae. aegypti* population. Conversely, the limited dissemination of the Old-World isolate in the Mexican mosquito population suggests that strain-specific genetic differences may influence vector compatibility. Elucidating these molecular determinants is essential for refining localized risk assessments and developing targeted strategies to mitigate ZIKV transmission.

## Supplementary Information

Supplementary Material 1. An excel file showing the blood meal and C_t_ values of *Ae. aegypti* samples used in the transcriptomics experiment

Supplementary Material 2. An excel file showing the quantification of Zika virusRNA loads in *Ae. aegypti *mosquitoes exposed to either Neotropics or Old-World ZIKV strain infectious blood meal

Supplementary Material 3. An excel sheet showing the read count and mapping statistics as well as the biotypes of RNA associated with each sample

Supplementary Material 4. An excel sheet showing the differentially regulated genes of mosquitoes fed on Old-World ZIKV strain–infected blood meal compared to a noninfectious control

Supplementary Material 5. An excel sheet showing the differentially regulated genes of mosquitoes fed on Neotropical ZIKV strain–infected blood meal compared to a noninfectious control

Supplementary Material 6. An excel sheet showing the gene ontology enrichment of mosquitoes exposed to Old-World ZIKV infectious blood meal to Mexican *Ae. aegypti* population

Supplementary Material 7. An excel sheet showing the gene ontology enrichment of mosquitoes exposed to Neotropical ZIKV infectious blood meal to Mexican *Ae. aegypti* population

Supplementary Material 8. A file of the full R workflow used to evaluate the effects of ZIKV infection on secondary blood-feeding success and egg production

## Data Availability

Raw sequences of RNAseq data have been deposited in the NCBI Bio Project database under accession number PRJNA1314310. Both ZIKV genomic sequences strains used in this study have been submitted to NCBI under the accession number PRJNA1426502.

## Abbreviations

ZIKV: Zika virus
RT-qPCR: Quantitative reverse transcription polymerase chain reaction
NS1: Non-Structural Protein 1
RNAseq: RNA sequencing
NS2A: Non-Structural Protein 2
PHEIC: Public Health Emergency of International Concern
JAK-STAT: Janus Kinase (JAK)-Signal Transducer and Activator of Transcription (STAT)
RNAi: RNA interference
ZIKV HOND: Zika Virus Honduras
ZIKV DAK: Zika Virus Dakar
