# Supplementary material for: Structural and nonstructural protein divergence correlates with strain-specific vector competence for Zika virus
Source: Parasit Vectors. 2026 Jul 13;19:434. doi: 10.1186/s13071-026-07569-w (PMC13647607; doi:10.1186/s13071-026-07569-w)
Supplement: Supplementary file 1 [file 13071_2026_7569_MOESM8_ESM.rtf]

# GLMM Analysis: Blood Feeding and Egg Production

# R version >= 4.2.0
# Required packages:
# glmmTMB
# lme4
# DHARMa
# emmeans
# tidyverse


# 1. Load Libraries


library(glmmTMB)
library(lme4)
library(DHARMa)
library(emmeans)
library(tidyverse)


# 2. Import Data


# Data format should include:
# Treatment (INF / NINF)
# Replicate (1,2,3,...)
# Fed
# NotFed
# Dead
# Eggs

data <- read.csv("egg_bloodmeal_data.csv")

# Convert to factors
data$Treatment <- factor(data$Treatment)
data$Replicate <- factor(data$Replicate)


# 3. Blood Feeding Analysis (Binomial GLMM)


# Create response as two-column matrix
data$TotalOffered <- data$Fed + data$NotFed

blood_model <- glmmTMB(
  cbind(Fed, NotFed) ~ Treatment + (1 | Replicate),
  family = binomial,
  data = data
)

summary(blood_model)

# Model diagnostics
blood_res <- simulateResiduals(blood_model)
plot(blood_res)

# Estimated marginal means
emmeans(blood_model, pairwise ~ Treatment, type = "response")


# 4. Egg Production Analysis


# First check dispersion
mean_eggs <- mean(data$Eggs)
var_eggs  <- var(data$Eggs)

cat("Mean eggs:", mean_eggs, "\n")
cat("Variance eggs:", var_eggs, "\n")

# If variance > mean -> use negative binomial

egg_model <- glmmTMB(
  Eggs ~ Treatment + (1 | Replicate),
  family = nbinom2,
  data = data
)

summary(egg_model)

# Diagnostics
egg_res <- simulateResiduals(egg_model)
plot(egg_res)

emmeans(egg_model, pairwise ~ Treatment, type = "response")


# 5. Model Outputs for Manuscript


# Extract fixed effects
summary(blood_model)$coefficients$cond
summary(egg_model)$coefficients$cond

# Likelihood ratio test
anova(blood_model)
anova(egg_model)


# 6. Optional: Plot Predicted Means


pred_blood <- as.data.frame(
  emmeans(blood_model, ~ Treatment, type = "response")
)

pred_egg <- as.data.frame(
  emmeans(egg_model, ~ Treatment, type = "response")
)

ggplot(pred_blood, aes(x = Treatment, y = prob)) +
  geom_point(size = 3) +
  geom_errorbar(aes(ymin = lower.CL, ymax = upper.CL), width = 0.2) +
  ylab("Predicted Blood Feeding Probability") +
  theme_classic()

ggplot(pred_egg, aes(x = Treatment, y = response)) +
  geom_point(size = 3) +
  geom_errorbar(aes(ymin = lower.CL, ymax = upper.CL), width = 0.2) +
  ylab("Predicted Egg Production") +
  theme_classic()


# End of Analysis
